# OLDER ADULTS’ SUBJECTIVE WELL-BEING EXPERIENCING THE EXERGAME “I AM DOLPHIN”

**DOI:** 10.1080/17482631.2023.2170013

**Published:** 2023-02-02

**Authors:** Brittany F. Drazich, Breanna M. Crane, Janiece L. Taylor, Sarah L. Szanton, Kyle D. Moored, Dana Eldreth, Omar Ahmad, John W. Krakauer, Barbara Resnick, Michelle C. Carlson

**Affiliations:** a School of Nursing, University of Maryland Baltimore, Baltimore, Maryland, USA; b Bloomberg School of Public Health, Johns Hopkins University, Baltimore, Maryland, USA; c School of Nursing, Johns Hopkins University, Baltimore, Maryland, USA; d School of Public Health, University of Pittsburgh, Pittsburgh, Pennsylvania, USA; e School of Medicine, Johns Hopkins University, Baltimore, Maryland, USA

**Keywords:** Exergames, activity, well-being, technology, virtual reality

## Abstract

The objective of this study was to understand older adults’ perceptions of the connections between an exergame intervention, “I Am Dolphin,” and their subjective well-being. Researchers conducted three focus groups with 14 older adults who participated in the exergame feasibility study. The semi-structured focus groups were transcribed, coded, and analysed using deductive and inductive techniques. Three themes were constructed related to playing the exergame and participants’ subjective well-being: 1) elevated mood (through scheduled activity, immersion, and socialization), 2) feelings of achievement (especially following frustration and competition), and 3) perceived cognitive or physical changes. These findings will help researchers better understand how exergames may relate to the well-being of older adults. Future investigators could use these findings to create and implement new exergame interventions.

## Background

The number of older adults (aged 65+) worldwide is expected to double by 2050 (0000). This growing population of older adults is not only concerned with how long they can live, but how well they can live. Focusing on the subjective well-being of older adults can provide a patient-centred approach to maintaining health over the lifetime for all individuals, regardless of their existing physical and mental health conditions (Forgeard et al., 2011; Friedman et al., 2019; Slade, 2010). According to Diener et al., subjective well-being consists of an individual’s emotions, both positive and negative, and their perceived life satisfaction (Diener et al., 1985, 2018). Research indicates that people with higher subjective well-being tend to have more friends, partake in healthier behaviours, experience better long-term physical health outcomes (Marcinko & Li, 2015), and live longer (Diener et al., 2018).

One innovative technology that has the potential to improve subjective well-being in older adults is exergames. Exergames or “exercise video games” (Oh & Yang, 2010) have shown promise in improving older adults’ physical function and balance (Alhasan et al., 2017; Neri et al., 2017; Skjæret et al., 2016). Research also indicates that exergames can improve depressive symptoms, psychosocial well-being, and health-related quality of life, which are concepts distinct from subjective well-being, which places emphasis on how an individual evaluates their own feelings and life satisfaction (Cacciata et al., 2019; Kahlbaugh et al., 2011; Li et al., 2016, 2017, 2018; Skevington & Böhnke, 2018; Xu et al., 2016). Only limited research has been conducted on exergames and the subjective well-being of older adults, indicating that playing exergames could increase their positive affect (Zheng et al., 2020). Additionally, most exergame research utilizes quantitative methods and does not focus on understanding older adults’ perceptions of how exergame play and health outcomes are related (i.e., the mechanism of action) (Diener et al., 2018; Gunst et al., 2022; Xu et al., 2016; Zheng et al., 2020).

Given this gap in the literature, our study aims to understand older adults’ perceptions of the connections between playing an exergame, “I Am Dolphin,” and their subjective well-being. Exploring this relationship is important because it can provide researchers and game designers with a deeper understanding into what aspects of exergames contribute to the improvement of well-being in older adults.

## Methods

This qualitative descriptive sub-study was conducted within the Stimulation With Intricate Movements (SWIM) study. The purpose of SWIM was to assess the feasibility and short-term cognitive and physical functional impact of an immersive and interactive exergame called “I Am Dolphin.” “I Am Dolphin” was created at the KATA Design Studio in the Neurology Department at Johns Hopkins School of Medicine and adapted for use in ageing adults. Although the game was initially designed for impairment reduction in upper extremities following cerebrovascular accident (Krakauer et al., 2018; Russell, 2015), it is applicable to older adults without a stroke history because it was designed to also improve a players’ overall cognitive, emotional, and physical health.

Through SWIM, 14 participants from an independent senior living facility played the exergame individually for one-hour sessions, three times a week, for five weeks. The objective of the game was to help Bandit, a dolphin, eat fish, jump, and avoid being eaten by sharks. While being monitored by a research assistant, the participants stood in front of a screen and moved Bandit with their dominant hand using a Kinect-based motion detection sensor. With a remote control in their non-dominant hand, the participants could flip Bandit’s fin to increase speed and/or stun sharks that were attempting to attack Bandit.

### Participants

The inclusion criterion for participants in this sub-study was participation in the SWIM study. Inclusion criteria for SWIM were: 1) English speaking, 2) 40 years of age or older, 3) absence of gross motor abnormalities that restrict ambulation (e.g., the ability to move around with or without using an assistive device), 4) ability to dedicate three hours per week for five weeks to gameplay, and 5) ability to give informed consent and understand the tasks involved. Exclusion criteria were: 1) cognitive impairment determined by a Montreal Cognitive Assessment (MOCA) (Freitas et al., 2013; Nasreddine et al., 2005) score ≤21, 2) red-green colour-blindness, and 3) an inability to perform upper limb exercises for an extended period (>30 minutes). All participants were recruited from a particular senior living facility.

All 14 SWIM participants who had participated in the intervention by the time of the focus group were recruited post-intervention via telephone for this sub-study. One of the 14 participants did not complete the SWIM intervention due to syncope from a chronic vestibular disorder but was still recruited for the focus group discussion. The study methods were approved by the Johns Hopkins University School of Public Health Institutional Review Board. All participants were fully informed prior to participation and signed an informed consent form according to the Declaration of Helsinki.

### Procedure

We determined that focus groups, rather than individual interviews, would spur discussion and idea exchange among the participants. We held three focus groups, with 4–5 participants in each focus group. We placed participants in groups that promoted homogeneity (e.g., pre-existing social networks, gender stratification) and comfort (Traynor, 2015). In an effort to spur participants to talk more openly and critically about the intervention, the individual selected to facilitate the focus groups (B.F.D.) was a research assistant who had no involvement in the SWIM study implementation. This female research assistant had previous experience facilitating focus groups. Another researcher (B.M.C.) was present to manage audio recordings and take field notes. Focus groups took place at the participants’ senior living facility, lasted approximately 75 minutes, and were held within 3–12 months of intervention activities for all participants.

The interview guide (Appendix A) used for the semi-structured focus groups concentrated on participants’ experiences with the exergame and their subjective well-being. At the beginning of each focus group, the facilitator informed the participants that the purpose of the focus group was to learn about their experiences playing the exergame, and thus, no answers could be considered “wrong.” To frame the subsequent discussion, the first interview guide question allowed the participants to provide their definition of well-being. Second, employing Diener et al.’s definition of subjective well-being, which consists of affective and judgemental components (Diener et al., 1985, 2018), the interview guide contained questions about the participants’ positive and negative emotions after playing the exergame and how playing the game related to feelings of life satisfaction and purpose. We recorded the interviews verbatim and a professional transcription agency, ProductionTranscripts, transcribed the audio (, 0000). The interviewer (B.F.D.) checked the transcriptions for accuracy.

### Data analysis

Two researchers (B.F.D., B.M.C.) analysed the focus group data using Maietta’s “sort and sift, think and shift” qualitative data analysis method, which consists of both deductive and inductive techniques (R. Q. S. Maietta, 2021; R. Maietta et al., 2021). First, B.F.D. and B.M.C. inductively coded one transcript, selected because it provided rich description and detail. This inductive coding was conducted through line-by-line close reading without considering any underlying frameworks or study objectives. Second, they listened to and read each focus group interview once. Next, they created a deductive codebook from their understanding of the literature and their focus group interview guide. This deductive codebook was based on main ideas and study objectives. B.F.D. and B.M.C. then met with the study principal investigator (M.C.C.) to compare their inductive codes with their existing deductive codebook and create a merged codebook. This merged codebook contained a code name, code abbreviation, a complete definition, when the code should be used, when the code should not be used, and an example of a corresponding quotation.

B.F.D. and B.M.C. used this single merged codebook to code all focus group transcripts. The coding process was iterative. At weekly meetings, they discussed if any new codes were needed, conducted reflexive dialogue, reviewed memos and field notes, and added new codes. Following coding, B.F.D. and B.M.C. met to compare coded data and created a qualitative data matrix to aid in the interpretation of the connections between findings. Using the matrix and the approach discussed by Merriam & Tisdell, they synthesized codes into categories and categories into themes (Merriam & Tisdell, 2016). They then searched for negative cases, or quotations that challenged their selected themes, and rival explanations, or alternative perspectives to understanding the data (Richards, 2016; Yin, 2016). They utilized F4analyse for the management of the qualitative data. F4analyse is a desktop-based coding software that offers quotation organization, continuous memo writings, and comparison of quotations across focus group transcripts (0000).

## Findings

The sample’s age ranged from 63–96 years old and the sample was predominantly female (*n* = 10) (Table 1). Three themes were constructed related to playing the exergame and subjective well-being: 1) elevated mood, 2) feelings of achievement, and 3) perceived cognitive or physical changes. The first theme, “elevated mood,” includes subthemes “elevated mood through activity,” “elevated mood through immersion,” and “elevated mood through socialization.” The second theme, “feelings of achievement,” includes two sub-themes “frustration leading to achievement” and “competition leading to achievement.” The third theme is “perceived cognitive or physical changes.”

**Table I.** Focus group participant demographics.

N=14	Mean (SD) or n (%)
Age	78.9 (8.7)
Age range	63-96
**Gender**	
Female	10 (71.4%)
Male	4 (28.6%)
**Race**	
White	12 (85.7%)
Black	2 (14.3%)
**Education**	
High School or Less	5 (35.7%)
> High School	9 (64.3%)
**Self-Reported Chronic Conditions**	
Hypertension	12 (85.7%)
Arthritis	11 (78.6%)
High Cholesterol	9 (64.3%)
Diabetes	3 (21.4%)

Note: Demographic characteristics collected at baseline SWIM evaluation.

### Definition of well-being

When asked to define well-being in their own words to frame the discussion, participants described a sense of feeling happy, comfortable, and loved. They said that well-being is feeling good about themselves or being satisfied with their lives. Participants also believed that a significant aspect of well-being was having intact physical and cognitive abilities to respond to the needs of life, accomplish their goals, and help others. Similarly, participant 1 defined well-being as “be[ing] able to pretty much handle what may come to you day-by-day.” Additionally, participants defined well-being as accepting limitations and adapting positively to change. They described the importance of resilience following negative life events and re-establishing life goals. One participant explained,

“I think that’s the whole thing with well-being is that you understand you have limitations or whatever, but you know, just keep ploughing forward.” Participant 3

The participants remarked on the importance of taking advantage of what life gives you.

### Elevated mood

The first theme from the focus groups related to playing the exergame and subjective well-being was mood improvement. Participants described how playing the exergame improved their mood through the activity of the game, feelings of immersion, and socialization, as will be described below.

#### Elevated mood through activity

The participants felt that the exergame improved their mood because they perceived that all activity improves mood. For example, participants discussed that both active (involving movement) and passive activities help them be productive and engage with life, making them happier. One participant remarked that playing the exergame elevated her mood because she felt her morning had been used wisely rather than wasted. Another participant commented

“ … being active engages the mind. Engaging the mind makes you feel more positive and feel better and that’s what I find about activities.” Participant 2

When asked about the duration of improved mood following gameplay, participants believed the changes were short term.

#### Elevated mood through immersion

Participants felt that the exergame improved their mood through feelings of immersion, or being completely absorbed in the game. The participants described this immersion as helping them forget their other life problems. One participant explained,

“Any time you’re playing the game and doing something out of the ordinary and it’s a fun thing to do … I mean the minute you stop and you start thinking about some other problems you have that can change in a hurry. But while you’re playing the game and if you’re enjoying then you’re just going to feel good.” Participant 8

Participants recalled playing “I Am Dolphin” as an “out of self” experience. They described being “so involved” that they talked to Bandit, the dolphin, or the sharks when playing. Two participants claimed that they felt maternal towards Bandit. Participants frequently mentioned that game session time elapsed quickly, and they were often surprised when it was time to end the session. When asked directly, no participants indicated that they were bored when playing the game.

*Elevated Mood Through Socialization*

Although “I Am Dolphin” is an individual player game, participants also felt that the exergame improved their mood through social aspects of playing the game. They described how verbally sharing their gaming experiences with their friends and family improved their mood. For example, participants remarked that they and other participants discussed “I Am Dolphin” at the dinner table at their senior living facility, comparing experiences and game progress. One participant expressed that playing the exergame helped her talk to her grandchildren about video games. She felt like she had developed a hobby to which her grandchildren could relate.

Additionally, participants in each focus group mentioned that their socialization with the research assistants who ran the game affected their mood. One participant said,

“You know, just coming down and socializing with [the research assistant] and talking I feel much better personally than I do if I just sit in my apartment by myself. Sometimes I have to make myself come down, but once I do, everything that I might worry about, my health, whatever, I forget about.” Participant 5

When asked if they would prefer to play “I Am Dolphin” collaboratively, rather than individually, each participant unanimously stated that they prefer individual play. They expressed concern that group play might become too competitive, with participants feeling that they would “hold other players back.”

### Feelings of achievement

The second theme from the focus groups related to playing the exergame and well-being is “feelings of achievement.” Participants looked forward to how much they would improve each session and how far in the game they would progress. Many participants described how they set personal gaming goals. A participant explained,

“I think everybody starting the game is going to improve at their own pace… Everybody is going to have a different path as to what they achieve.” Participant 2

The participants used words such as “achievement,” “accomplishment,” and “satisfaction” when discussing conquering their goals. One participant mentioned that she found it especially helpful to have the instant gratification of Bandit on the screen, offering congratulations once she completed a level. Participants expressed that on days they played the exergame they felt that they “accomplished something that day.” They were also satisfied at the end of the intervention, feeling that they “saw something to the end” and did “not give up.” Lastly, participants described how feelings of frustration or competition often led to feelings of achievement, as will be described below.

#### Frustration leading to feelings of achievement

Many participants discussed that the frustration experienced while playing the exergame was a precursor to eventual feelings of achievement. All focus groups referred to being stuck on the same level, feeling like they would not progress in the game, or having difficulty with particularly aggressive sharks. One participant stated,

“I remember getting frustrated when I was on a particular level and I absolutely could not kill those fish—or sharks. I couldn’t catch them, and it just went on and on.” Participant 1

During conversations about frustration, participants expressed annoyance that the sharks would not stay stunned very long and that Bandit, the dolphin, seemed at times slower than the sharks. They also expressed frustration when the sharks would be “off-screen” and become difficult to find. Discussions of frustration were often followed by the participants expressing how they felt satisfied or accomplished when they eventually conquered their goal. For example, one participant stated,

“That was frustrating when I had to stay at the same level … That got very frustrating, and then all of a sudden when you didn’t think you were going to do it, you just moved right on up to the next level. And that was very rewarding.” Participant 10

During particularly frustrating levels and situations, the participants expressed that their eventual accomplishment was more satisfying.

*Competition Leading to Feelings of Achievement*

Participants also discussed that the competition experienced while playing the exergame was a component of eventual feelings of achievement. One participant commented,

“I wanted to get the sharks. And I didn’t care about them, you know? I wanted to get them! <laughter> .” Participant 12

Another participant described

“the feeling of accomplishment when you finish especially one of the harder levels with those blankety-blank sharks.” Participant 6

Some participants expressed that they were not naturally competitive, but that playing the exergame brought out their competitive side. One participant described strategizing during the week to try new techniques at their next gaming session. This participant said playing the exergame was like working against themselves and working towards self-betterment.

### Perceived physical or cognitive changes

#### Physical changes

Some of the participants expressed a perception that playing the exergame affected them physically. The participants described how playing the exergame got them out of their apartment and “down there to do a physical exercise.” In one example, a participant who had existing unilateral weakness felt that playing the exergame brought back some of the movement to the injured side. This participant remarked,

“ … it just helped me make some connections in my brain that had gotten lazy, shall we say? Because I knew before I couldn’t use the arm at all.” Participant 14

However, many participants also described experiencing short-term increases in pain and fatigue following gameplay. Participants commented on minor pain in their back, hip, arm, shoulder, and knees. These participants conveyed that their ability to take breaks when they perceived that they were needed was crucial to the intervention. One participant expressed concern that participants might

“push themselves so hard because they get so aggressive trying to kill sharks, that that might actually damage themselves.” Participant 14

*Cognitive Changes*

Participants varied in their opinions regarding if playing the exergame affected them cognitively. Some participants did not believe that their cognitive performance changed. For instance, one participant explained that after they had mastered the basics of the game, it did not require much “brainpower.” They suggested more cognitively challenging tasks in later levels. Other participants did feel that playing the exergame made them “smarter” or “added something to [their] mental acuity.” One participant described the cognitive skills that took place while playing:

“Using your brain, because you had to tell your finger, ‘You got to do this to catch that fish … I can catch them off-guard.’ So you was thinking through this process. I don’t even think people realized that you know you was actually thinking.” Participant 12

## Discussion

The purpose of this study was to understand older adults’ perceptions of the connections between playing an exergame, “I Am Dolphin,” and their subjective well-being. The participants’ definition of well-being as feeling happy, having intact cognitive and physical abilities, handling their affairs and helping others, and responding well to difficulties, was similar to past research on the subjective well-being of older adults (Bowling et al., 2003; Diener et al., 2018; Douma et al., 2017). This definition helped offer context for exploring the objective of this study. Three themes were constructed related to playing the exergame and wellbeing: 1) elevated mood, 2) feelings of achievement, and 3) perceived cognitive or physical changes.

The first theme related to the exergame and well-being was “elevated mood.” The current literature supports the finding that activity, such as fitness-based activity and behavioural activation, improves the mood of older adults (Kanter et al., 2010; Klil-Drori et al., 2020; Luoto et al., 2018; Miller et al., 2020; Perez-Sousa et al., 2020). Additionally, given that humans’ relationship with animals can provide positive physiological and psychological health benefits (Aarskog et al., 2019; Peluso et al., 2018), perhaps the use of an animal avatar for this exergame provided a unique immersive and emotional experience for participants that led to mood improvement. The discovery that the exergame affected their mood through the “social aspects” of playing the game was surprising because “I Am Dolphin” is an individual player game. The participants explained that they perceived social effects through interacting with the research assistants who ran the game, which is an effect from the study itself rather than engagement with the exergame. Participants also perceived social effects through discussing the game with family and friends.

Although participants felt improved mood due to the social aspects of playing “I Am Dolphin,” all participants unanimously recommended against making the game collaborative, expressing concern that they would hold their teammates back. Considering that the participants described well-being as having the cognitive and physical abilities to achieve goals and help others, they might have been concerned that they would not have the abilities needed to contribute to a team given a group format. Although the participants of this study verbalized their preference for individual exergame play, it is important to note that previous research indicates that exergames can foster a sense of connection and decrease loneliness among older adults (Maillot et al., 2012; Mayr et al., 2012; Ogawa et al., 2016). Lastly, as participants perceived only short-term improvements in mood from exergame play, future exergame researchers might explore how to generate more long-lasting effects, such as increasing home exergame accessibility.

The second theme related to the exergame and well-being was feelings of achievement following frustration and competition. Their positive view towards self-competition is interesting, considering that past research indicates that older adults diminish in competitive orientation as they age (Mayr et al., 2012). Perhaps participants in this study perceived the competition more positively because they were playing against a game rather than a person. If the participant did not perform well, they did not embarrass themselves or disappoint anyone.

The third theme related to the exergame and well-being was “perceived cognitive or physical changes.” This theme reflects participants’ emphasis on having intact cognitive and physical abilities to respond to the needs of life. The participants perceived that playing the exergame was physical activity, and experienced positive feelings leaving their apartment and taking care of their bodies. Similarly, previous literature indicates that exergames can improve mobility, balance, and physical function (Alhasan et al., 2017; Neri et al., 2017; Skjæret et al., 2016). Conversely, participants reported experiencing increased short-term pain from playing, alleviated by rest periods. This finding suggests that all older adult exergame interventions should include the ability to sit and rest during gaming. Participants varied in their opinions regarding if the exergame contributed to cognitive improvements. Past quantitative research on the effects of exergaming on cognitive function in older adults indicates improvement in executive functioning and processing speed (Maillot et al., 2012; Ogawa et al., 2016).

In addition to the participants’ own definitions of well-being, this study also utilized Diener et al.’s proposed definition, which consists of a combination of an affective component and a judgemental component (Diener et al., 2018). The first theme regarding the exergame and mood reflects the affective (emotional) component of well-being (Diener et al., 2018). The second theme regarding the exergame and achievement, especially feelings of satisfaction, reflects the judgemental (life satisfaction) component of well-being (Diener et al., 1985). Although the third theme regarding perceived cognitive or physical changes does not reflect the affective or judgemental components of Diener et al.’s definition of subjective well-being, they were still included in our findings because the participants expressed the importance of intact abilities to their well-being in the focus groups (Rowe & Kahn, 1997).

### Limitations and strengths

Limitations of this study include the focus group format and limited sample size. Although the focus group format was purposely selected to promote discussion and idea exchange among the participants, the participants could have felt swayed by the opinions of their peers (i.e., groupthink) (McLeod & Feller, 2019), and not divulged their true feelings. The sample of participants was limited to those who participated in the SWIM study. Additionally, these participants were recruited from a particular senior living facility, which can limit the transferability of findings. The experiences of these participants might not represent the rich and diverse perspectives of all community dwelling older adults. Lastly, while no new codes arose in the last focus group, we were unable to confirm saturation through further focus groups (Walker, 2012).

This is one of the first studies to qualitatively explore the connections between an exergame and well-being. This study stands out from other exergame literature because it explores an exergame that was created to improve the cognitive and physical health of its players, rather than for commercial uses. Reflecting senior living facilities in the United States, our sample was predominantly female. The sample’s age ranged from 63–96 years old and we included participants who reported a wide variety of physical limitations and medical comorbidities (e.g., hypertension, arthritis, and diabetes), who are commonly excluded from exergame studies (for Community Living A, 2020; Roberts et al., 2018; Spitzer et al., 2004). Despite these physical limitations, the participants, with the exception of one individual, were able to complete the intervention.

## Conclusions and implications

Our study demonstrates that older adults perceive that playing an exergame, “I Am Dolphin,” improved their well-being through elevated mood, feelings of achievement, and in some cases, perceived physical/cognitive changes. Considering the primarily white participant sample for this study, future exergame researchers should consider recruiting more racial and ethnically diverse older adult participants. These findings contribute to the literature by providing researchers and game designers with a deeper understanding into what aspects of exergames are important to improving the well-being of older adults. For example, future game designers can consider using immersive game environments that might help improve the players’ mood. They might also consider methods to optimize the type of competition that older adults find appealing in exergames, such as playing against themselves or a computer. Investigators can ensure rest periods to help alleviate participant fatigue or pain among older adult participants. As older adults are the fastest growing cohort, health researchers should take advantage of innovative technologies that can improve not only how long they live, but also how well they live.

## Appendix A. Interview guide

1. What does well-being mean to you?

Based on your explanation of well-being you just gave, how did “I Am Dolphin” relate to your well-being?

2. After playing “I Am Dolphin,” what is your mood like the rest of the day?

3. Describe how on days you played “I Am Dolphin” you were more or less active for the rest of the day.

Do you feel like this change in activity lasted long-term?

4. Describe how activity (like gardening, singing, talking on the phone, etc., or even “I Am Dolphin”) and well-being relate in your life.

5. Describe if and how “I Am Dolphin” may have contributed to you feeling a sense of purpose?

6. Describe how playing “I Am Dolphin” made you feel more or less in control or independent.

7. What is your mood like when you are more active?

8. Describe any feelings of satisfaction you experienced during “I Am Dolphin” sessions or in between “I Am Dolphin” sessions.

Is there anything you would like to share that you did not have the chance to share already?
